# Analyzing drama metadata through machine learning to gain insights into social information dissemination patterns

**DOI:** 10.1371/journal.pone.0288932

**Published:** 2023-11-30

**Authors:** Chung-Ming Lo, Zih-Sin Syu

**Affiliations:** Graduate Institute of Library, Information and Archival Studies, National Chengchi University, Taipei, Taiwan; First Technical University, NIGERIA

## Abstract

TV drama, through synchronization with social phenomena, allows the audience to resonate with the characters and desire to watch the next episode. In particular, drama ratings can be the criterion for advertisers to invest in ad placement and a predictor of subsequent economic efficiency in the surrounding areas. To identify the dissemination patterns of social information about dramas, this study used machine learning to predict drama ratings and the contribution of various drama metadata, including broadcast year, broadcast season, TV stations, day of the week, broadcast time slot, genre, screenwriters, status as an original work or sequel, actors and facial features on posters. A total of 800 Japanese TV dramas broadcast during prime time between 2003 and 2020 were collected for analysis. Four machine learning classifiers, including naïve Bayes, artificial neural network, support vector machine, and random forest, were used to combine the metadata. With facial features, the accuracy of the random forest model increased from 75.80% to 77.10%, which shows that poster information can improve the accuracy of the overall predicted ratings. Using only posters to predict ratings with a convolutional neural network still obtained an accuracy rate of 71.70%. More insights about the correlations between drama metadata and social information dissemination patterns were explored.

## Introduction

Drama is seen as a form of storytelling in which human relationships are usually performed by actors through dialogue or action. There are many types of drama, which can be recorded and edited into forms for radio, cinemas, and television (TV) to convey narrative development through different kinds of performance. TV drama combines the advantages of radio dramas and movies to satisfy the human need for stories in an unprecedented way [1]. People may have a tendency to watch TV dramas when they are anxious and want to escape from reality. The entertainment function of TV dramas has become a recovery strategy in the face of stress [2]. COVID-19 has caused more people to shift their entertainment from outdoor to indoor activities, and watching drama has become a more popular way to relieve stress.

The plot of a drama is often based on real life so that the audience can empathize with the characters, and drama can cause audiences to subconsciously reveal shared values and attitudes in society [3]. By watching drama, one can not only observe regional or national culture and identify local social phenomena but also notice changes in mainstream values shared across society over time [4]. Compared with other countries, Japanese drama focuses more on the family or workplace relationships of the characters and depicts explorations of all the social situations in daily life. Suzuki [5] investigates the dominant ideologies in the NHK morning serial drama (asadora), Massan, including frame issues of language, courtship, race, gender, family, and nationalism. Mandujano-Salazar [6] analyzes Japanese dramas in which single women who are over 30 years old and childless reflect the values of the new generation of women and their resistance to traditional stereotypes.

However, when TV dramas invest a lot of money into production and ratings are extremely low or not as expected, it will not only affect the TV station’s advertising revenue but may even cause the station to end the show early in order to stop the damage immediately. The close relationship between the TV drama production budget and ratings makes ratings an indicator of the commercial success of a TV drama. According to Zenith [7], the global TV advertising market will be worth more than $150 billion in 2020 and is forecast to continue to grow thereafter. To evaluate and measure the effectiveness of their investment, advertisers often consider ratings as a decisive indicator of ad placement, and they believe that ratings are currently correlated with ad exposure [8]. To avoid low ratings, it is important to use objective TV drama data to analyze and predict ratings and understand market demand to plan TV dramas that meet audience needs. This study thus quantified drama metadata, including broadcast year, broadcast season, TV station, broadcast day of the week, broadcast time slot, genre, screenwriters, status as an original work or sequel, cast and facial features in posters. These drama features were then combined in machine learning classifiers to predict drama ratings [9]. By analyzing the contribution and statistical distribution of different features, more information on social information dissemination patterns can be explored.

By taking into account the audience’s regular viewing time slots, preferred TV stations, and preferred genres and actors, producers can allocate time slots more effectively and tailor content and promotion strategies to better suit the audience. This can result in higher drama ratings, increased advertising revenue, and a better viewing experience for the audience, all while reducing marketing costs and creating a positively reinforcing cycle of success.

### Related works

Bristi, et al. [10] used machine learning to predict audience ratings on the Internet Movie Database (IMDb). They collected information from 242 movies released in Hollywood in 2018 using year, director, screenwriter, actor, actress, genre, country, and production company as features in bagging, random forest, k-nearest-neighbors (KNN), J48 decision tree, and naïve Bayes classifiers. User ratings were classified into four labels: flop, below average, average, and hit. Each classifier reached over 90.00% accuracy, while random forest had the highest accuracy of 99.20%. Sharda et al. [11] used a multilayer perceptron to predict the box office performance of a movie before its release. From 1998 to 2002, 834 movies were collected and classified into nine labels from low to high. The features included the Motion Picture Association of America movie rating, competition time slot, star value (depending on the budget of the movies in which the actor has acted), genre, technical effects, sequels and number of screens. The accuracy reached 75.20%. Lee et al. [12] used decision tree, KNN, linear regression, and ensemble methods to predict movie box office returns. From January 2014 to May 2016, box office data for the first 3 weeks after the release of 1439 movies were predicted using features including movie awards, movie ratings, sequels, release dates, genres, production countries, and word of mouth (recommendation or not, number of reviews, ratings, etc.). The decision tree using the ensemble method outperformed the linear regression using ensemble methods.

In a study predicting TV program ratings, Danaher and Dagger [13] collected data from 70 channels during the period from January 2004 to June 2008 and used a nested logit model to predict the overall audience choice of each program. All programs were classified into four categories: light content (e.g., reality shows and comedies), heavy content (e.g., documentaries and news), sports, and movies. Compared with previous studies, the result reduced the estimate error by 1.08 rating points. In the literature [14], the authors used linear support vector regression and random forest to predict the ratings of the first episode of 678 Japanese TV dramas from 2008 to 2015. The features included TV stations, broadcast time slots, cast, related staff, and actors’ topicality. The resulting Pearson correlation coefficient between the actual and predicted ratings was 0.84.

Several studies have used image features for prediction. Huang, et al. [15] created a dataset of 44 trailers for drama, thriller, and action movies and used the shot length, color variance, motion content, lighting, and moving effects to predict genres. The accuracy was 73.33%. Kundalia et al. [16] built a prediction model using a total of 2500 movie posters from 1902 to 2019 for each of 12 movie genres, including action, adventure, animation, comedy, crime, drama, fantasy, horror, suspense, romance, science fiction, and thriller. Based on the predictions made by a convolutional neural network (CNN), the accuracy of classification was 84.82%. Zhou et al. [17] used 3807 movies on IMDb as a dataset and achieved 63.15% accuracy in predicting ticket sales by applying a CNN to movie posters. Combining the features of movie posters, genres, running time, production budget, actors, directors, movie reviews, and movie ratings, a deep neural network generated an average percent hit rate of 88.60%. This study proposed the use of CNN to extract facial information from posters and combine it with various factors such as broadcast year, season, TV stations, day of the week, time slot, genre, screenwriters, status as original work or sequel, and the cast in machine learning classifiers that predict ratings. A CNN is a powerful deep learning architecture that can extract highly accurate facial information in great detail. This study collected a larger amount of data on Japanese TV dramas than previous studies, providing a more comprehensive analysis of the entire picture. This is especially important given the impact of recent social media trends and COVID-19 on watching habits, which should be explored to better understand the latest dissemination patterns.

## Materials and methods

**Fig 1:**
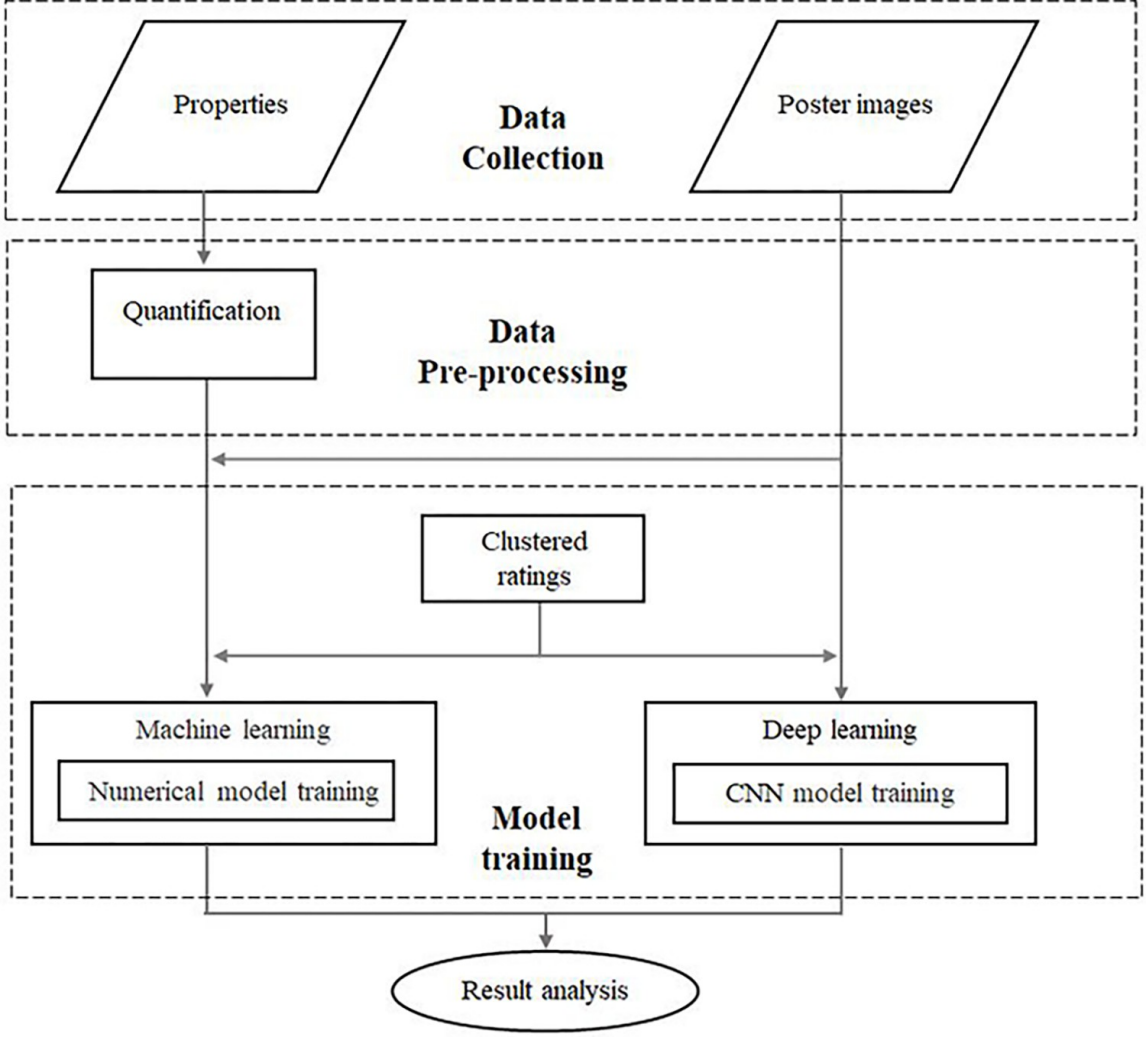
Flowchart of the proposed analysis.

Fig 1 shows the flowchart of the proposed analysis. First, the metadata and poster images of TV dramas were collected. The metadata were then quantified and normalized to serve as classification features. The poster images were not only used to extract facial information for combination with the metadata features but also directly used alone to predict the ratings for comparison. In the ratings prediction, high ratings and low ratings were defined using clustering and used as the classification labels in model training.

### Data collection

The datasets provided with the study include the drama metadata as described below and the corresponding python codes processing the data to be features used in classifiers. The data was generated from the survey and read from the website and the code was written by us. The collection and analysis method complied with the terms and conditions for the source of the data.

#### TV drama metadata

TV dramas broadcast by Japanese commercial TV stations during prime time were the experimental targets. A total of 800 TV dramas from 2003 to 2020 were collected. Prime time is the time period when most people watch or listen to TV or radio programs [18] and is generally considered to be between 19:00 and 23:00 in Japan. The ratings of dramas broadcast during this time period reflect a majority choice from the audience for programming. Drama metadata were obtained from Audience Rating TV, a database dedicated to collecting ratings of Japanese TV dramas, and TV-log, a TV drama rating website. The TV rating values were from Video Research, a Japanese TV ratings company.

*I*. *Broadcast year*, *season*, *day of week*, *broadcast time slot and TV station*. Time spent watching TV dramas is closely related to audience leisure and entertainment habits. The broadcast time thus determines the audience’s intention to watch them. Japanese TV dramas are broadcast for 3 months, and most dramas premiere in January, April, July and October, so the broadcast period can also be called broadcast seasons. For example, a drama broadcast in January is in the "winter time slot", and a drama broadcast in April is in the "spring time slot". In Japan, the time of the broadcast is often combined with the day of week to order the time slot of a TV drama. For example, a drama broadcast on Monday at 9:00 is called "Getsuku (月9)", and a drama broadcast on Wednesday at 10:00 is called "Suijyu (水10)". Sometimes the time slot is also associated with a specific TV drama topic; for example, most of the TV dramas that are broadcast on "Getsuku" are in the romance genre.

The TV station features included in the dataset are Fuji TV (CX), Nippon TV (NTV), Asahi TV (EX), and TBS. Compared to NHK, which obtains its production budget from the Japanese government, commercial TV stations are more regular and stable in announcing their ratings to be accountable to the advertisers who pay them. The development strategies of TV stations may affect the thematic preferences and budgets of TV dramas, and these differences may be reflected in the ratings performance.

*II*. *Genre*. Originally, the TV drama genre features were from the "TV-log" website and included 23 TV drama genres. Six genres, crime, science fiction, adventure, documentary, violence, and horror, were merged into other existing genres with similar definitions in the dataset, e.g., period dramas belonged to the history genre. Table 1 shows the remaining 17 genres.

**Table 1:** Distribution and definition of 17 genres.

Genre	Number	Definition
Medical	70	Related to the medical field
Police	100	Police officers investigate and solve crimes
Sports	17	Related to sports
Youth	63	Focuses on childhood or school life
Comedy	42	Meant to be funny
Mystery	54	Mystery or puzzle
Romance	126	Focuses on the process of romance
History	16	Time before the Meiji era
Law	24	Related to litigation scenarios
Politics	4	Related to the field of political themes.
Occupation	82	Focuses on a specific occupation, or what happens in the workplace
Fantasy	16	Existence of supernatural beings or lifeforms
Family	89	Related to the theme of family relationships
Emotion	46	Depicts human feelings and emotions
Suspense	35	The sense of unease and tension
Action	7	Focuses on fighting or actions
Life	9	Depicts the life of the main character

*III*. *Cast and screenwriter*. Treme [19] stated that the influence and popularity of a celebrity can affect the box office revenue of a movie, and the more famous the actor, the more likely he or she is to choose a high-quality script and production team. This phenomenon may also occur in the casting of television dramas. Similarly, well-known screenwriters also have a certain degree of star power to audiences. The basic setting and integrity of the story are closely related to the scripts produced by the screenwriters.

*IV*. *Original work and sequels*. Chang and Ki [20] indicated that filming adaptations of familiar comics, novels and other works or making sequels to well-known existing TV dramas and movies has a brand advantage for the producer, which can make the works more likely to attract attention before broadcasting and reduce the marketing cost for the spin-off drama. Familiarity may even become an incentive for the audience to watch.

#### Poster images and features

**Fig 2:**
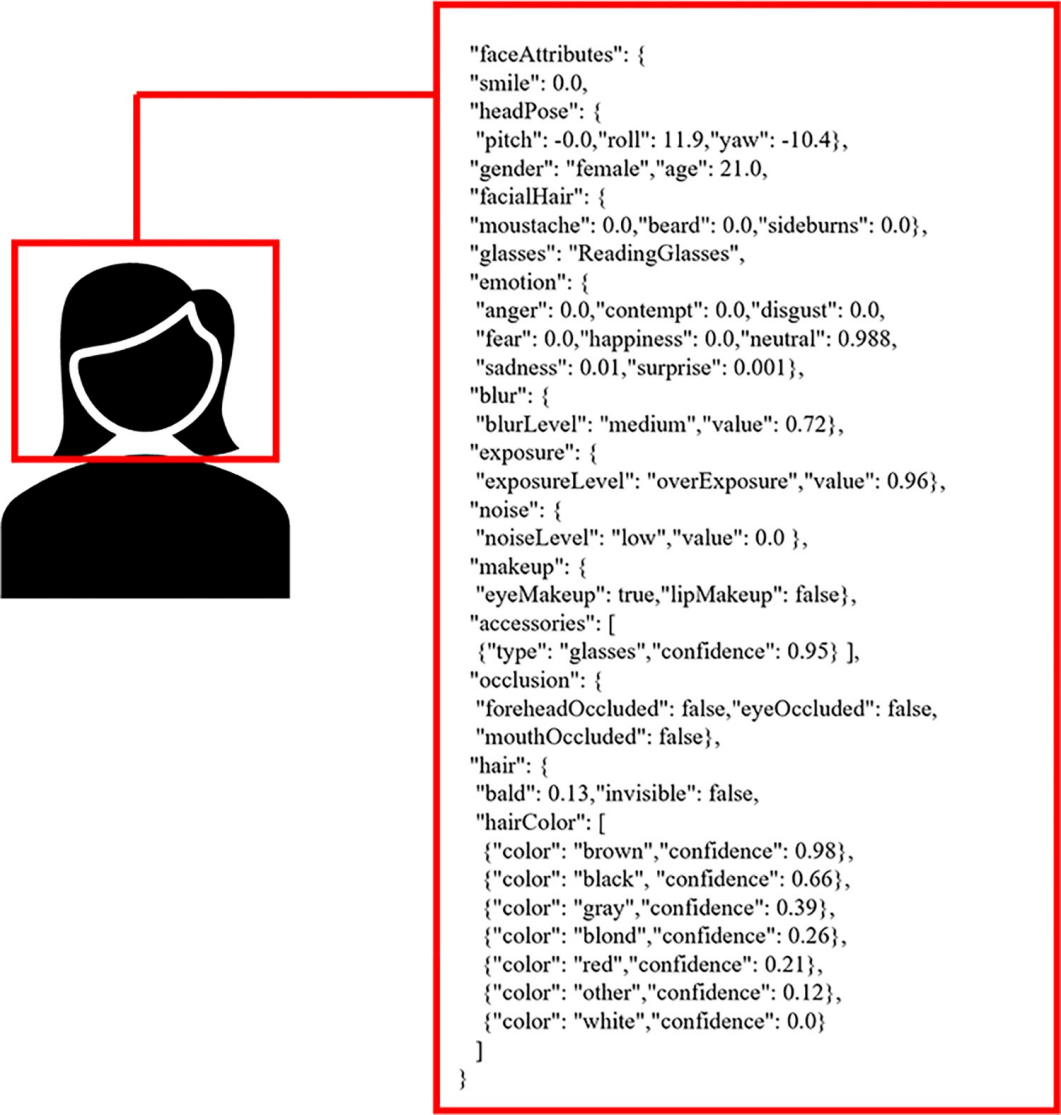
The detected faces and features.

For each drama, the representative poster image was determined according to the cover of the official DVD. If the drama was not released on DVD, the poster provided by the official website was chosen. Most of the actors appearing on the posters of TV dramas are the main characters who frequently appear in the stories. The gender, age, makeup and emotion of the actors on the posters are factors affecting the ratings. Microsoft Azure Face was applied to extract facial information from 800 TV drama posters, as shown in Fig 2. By averaging the metadata of multiple faces in a poster, nine types of features were selected: the number of detected faces, the average smile level, the maximum age, the minimum age, the average age, the proportion of females, the proportion of glasses, the number of hair colors and the number of emotions.

### Preprocessing

**Fig 3:**
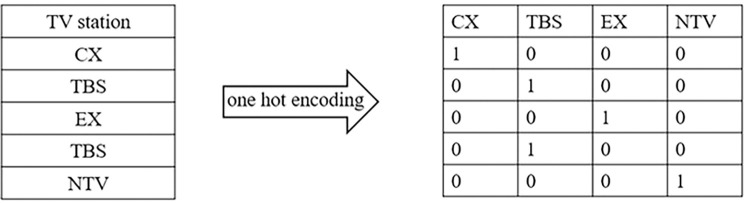
New TV station features after one-hot encoding.

Discrete features with a temporal order were normalized by label encoding [21]. The value between 0 and the number of categories minus 1 is used to convert the vector of categories into encoded integer representations. For example, the year feature from 2003 to 2009 was encoded into a number from 0 to 6, increasing the readability of the classifier for this feature. Nominal discrete features without sequential relationships were normalized using one-hot encoding, as used by Danaher et al. [13], to generate more feature fields according to the various possible option values in the original feature. In each new feature field, binary values were created to show the existence of an option. A sparse matrix or dense array is thus returned [22], as shown in Fig 3.

**Fig 4:**
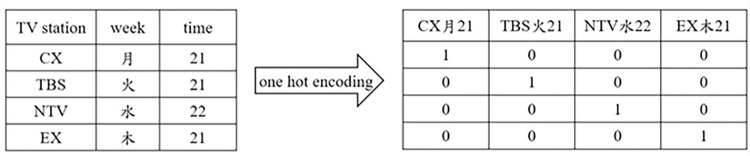
Scheduled slot as an integrated feature by one-hot encoding.

Considering that some scheduled time slots already have word-of-mouth reputations and can consistently attract a group of loyal audiences, an integrated feature “scheduled slot” combining the TV stations, days of the week, and broadcast time slot was also generated using one-hot encoding, as shown in Fig 4.

**Fig 5:**
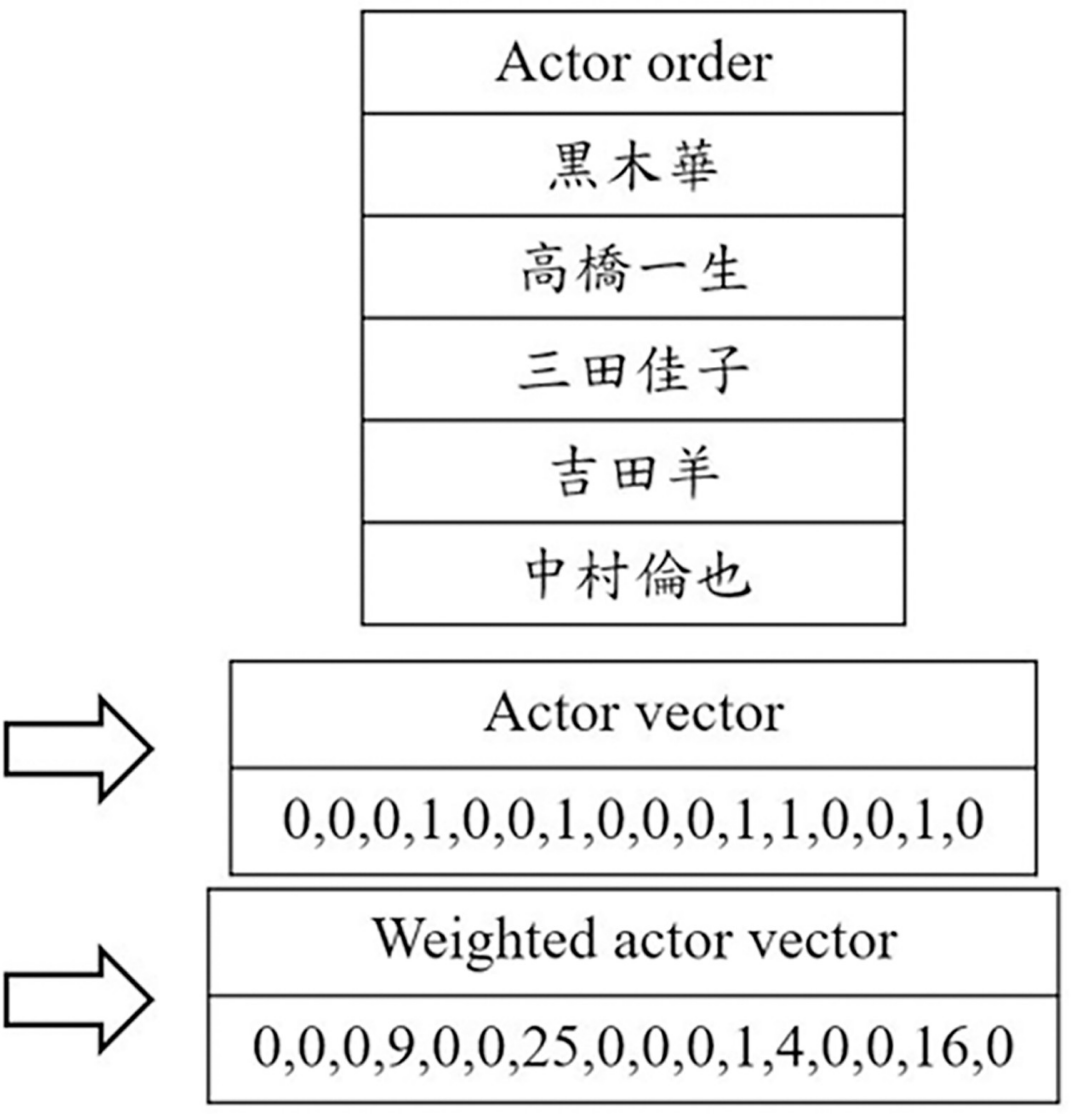
The normalized and weighted cast features.

Another feature based on one-hot encoding was the cast. A total of 957 different actors were identified in the collected dramas. The cast list of a drama is presented by a series of 0s or 1s. In particular, the participation ratio of actors, i.e., the ranking order of actors in the Drama Database was weighted by squared values. In Fig 5, the original cast list was vectorized and weighted to emphasize the actors’ order.

### Numerical prediction model

Using a well-known rating score from Video Research, the covered population was the Kanto area, which has approximately 43 million people, accounting for 34.40% of Japan’s total population. The information about this region is also used by most mainstream news outlets, and the area has the largest population in the entire survey area. K-means with a silhouette coefficient [23] was then used to have an objective benchmark, which determined that the best number of clusters to separate the rating scores was two, into high or low. K-means is a partitional clustering algorithm that attempts to find k nonintersecting clusters, and the centroid in the cluster is the cluster center. Supposing there is a dataset D containing n data points in Euclidean space, the partitional clustering algorithm divides the data into k clusters, denoted as *C*_1_, …, *C*_*k*_, where *C*_*i*_ is a cluster existing in dataset D. The sum of squared errors between the points and the centroid is calculated to identify the data points that are close to each other and in the same cluster. As shown in Eq (1) [24], *di* represents the centroid of cluster *Ci*, *p* is the data point within cluster *Ci*, and *E* is the sum of squared errors of all points in the dataset.


$$E=\sum_{i=1}^{k}\sum_{p\in C_{i}}dist{\left(p,d_{i}\right)}^{2}$$ (1)

Centroids are randomly selected first. Then, each data point is assigned to the closest centroid. By calculating new centroids in clusters after the introduction of new data points that are assigned to each centroid, the clustering is updated. After multiple iterations, the process stops when the centroid does not move significantly. The silhouette coefficient compares the intracluster tightness with the separation between the remaining clusters to evaluate the effectiveness of clustering and to suggest the appropriate number of clusters [25]. As shown in Eq (2) [23], a data point is randomly selected as *i*, and *A* is the cluster to which *i* belongs. When Cluster *A* contains objects other than *i*, *a(i)* is the average distance of data point *i* from other data points belonging to Cluster *A*. When there is another Cluster *C* other than *A*, *b(i)* is the minimum average distance between data point *i* and all data points of Cluster *C*. When *a(i)* is smaller, it means that Cluster *A* is more compact; when *b(i)* is larger, it means that Cluster *A* is more separated from Cluster *C*. When the silhouette coefficient *s(i)* is larger, it means that the intracluster variation *a(i)* is smaller than the minimum intercluster variation *b(i)*, indicating that the result of clustering is better.


$$s(i)=\frac{b(i)-a(i)}{max\{a(i),b(i)\}}$$ (2)

Using the clustering result as the classification target, four numerical classifiers, including the naïve Bayes classifier (NB) [26], artificial neural networks (ANN) [27], support vector machine (SVM) [28], and random forest (RF) [29], were used to evaluate the proposed TV drama features. NB assumes that the features of the samples are independent of each other and calculates the probability of the occurrence of each category to identify which has the highest probability for classification. The advantage of the NB is that it is efficient, and because of the nature of the probabilities used, it is robust to missing values and noise in the sample. ANNs can generally be divided into three parts: input layer, hidden layer, and output layer. The input layer is responsible for receiving features from the dataset. Its values are passed to the hidden layer, which consists of several neurons and uses linear regression to combine features. The final output layer is responsible for generating and presenting the final classification results, which are generated by the execution of the previous layers of neurons. SVM finds the maximum margin between categories and derives the most generalized hyperplane for classification as the decision boundary. The data points located at each category boundary are called support vectors. When the data are nonlinearly separable, the kernel function can be used to nonlinearly map the data to a higher dimensional space to generate nonlinear boundaries and further classify nonlinear datasets. RF is a combinatorial classifier composed of multiple decision trees. Using bootstrap-aggregating and the splitting criterion of decision trees, random samples are repeatedly selected from the training dataset to create a large number of fully grown decision trees. The final prediction is determined by voting or averaging the data.

### Image prediction model

Considering that TV drama posters usually contain elements related to the story content and serve as a medium to advertise to the public, the composition of the posters is analyzed to find the correlations with ratings. Poster compositions are complex due to the presence of various elements, such as colors, lines, object arrangements, backgrounds and high-level suggestive patterns. To better interpret image characteristics, CNNs including AlexNet [30], Inception V3 [31], ResNet 152 [32], and DenseNet 201 [33] were used to connect the correlations between image compositions and ratings. By using convolutional layers, CNNs can automatically perform a variety of image feature extractions, combining low-level features with high-level features in layer-to-layer connections. Typical CNN architectures consist of a convolutional layer, pooling layer, and fully connected layer. Convolutional layers consist of multiple convolutional kernels that perform convolutional operations on the input images to generate different feature maps. Pooling layers aim to preserve the most dominant features while reducing the size of the feature map and are usually located between the two convolutional layers. Fully connected layers are usually located in the last part of a CNN architecture to compute the final classification results.

## Results

### Statistical distribution of feature values

**Fig 6:**
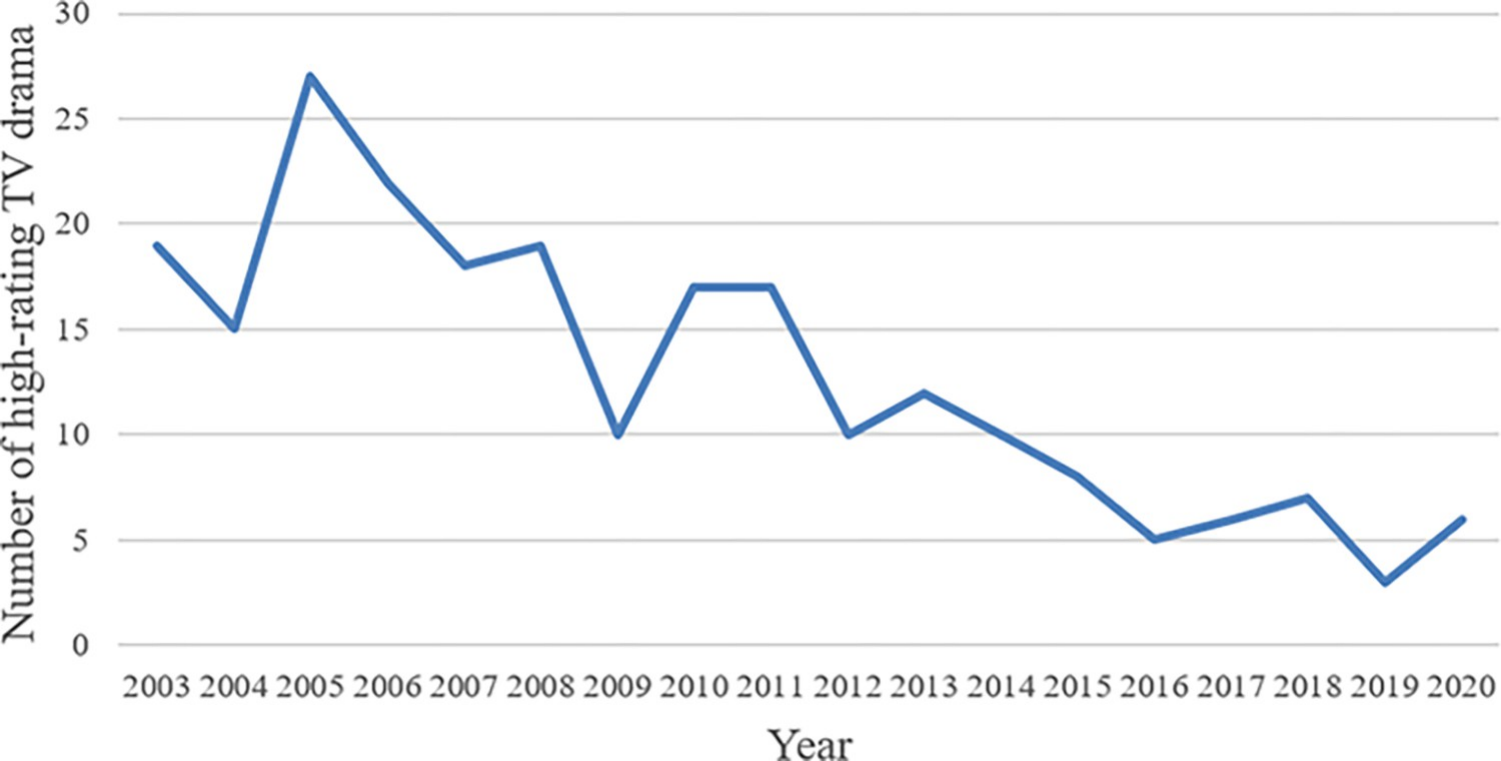
The number of highly rated dramas by year.

**Fig 7:**
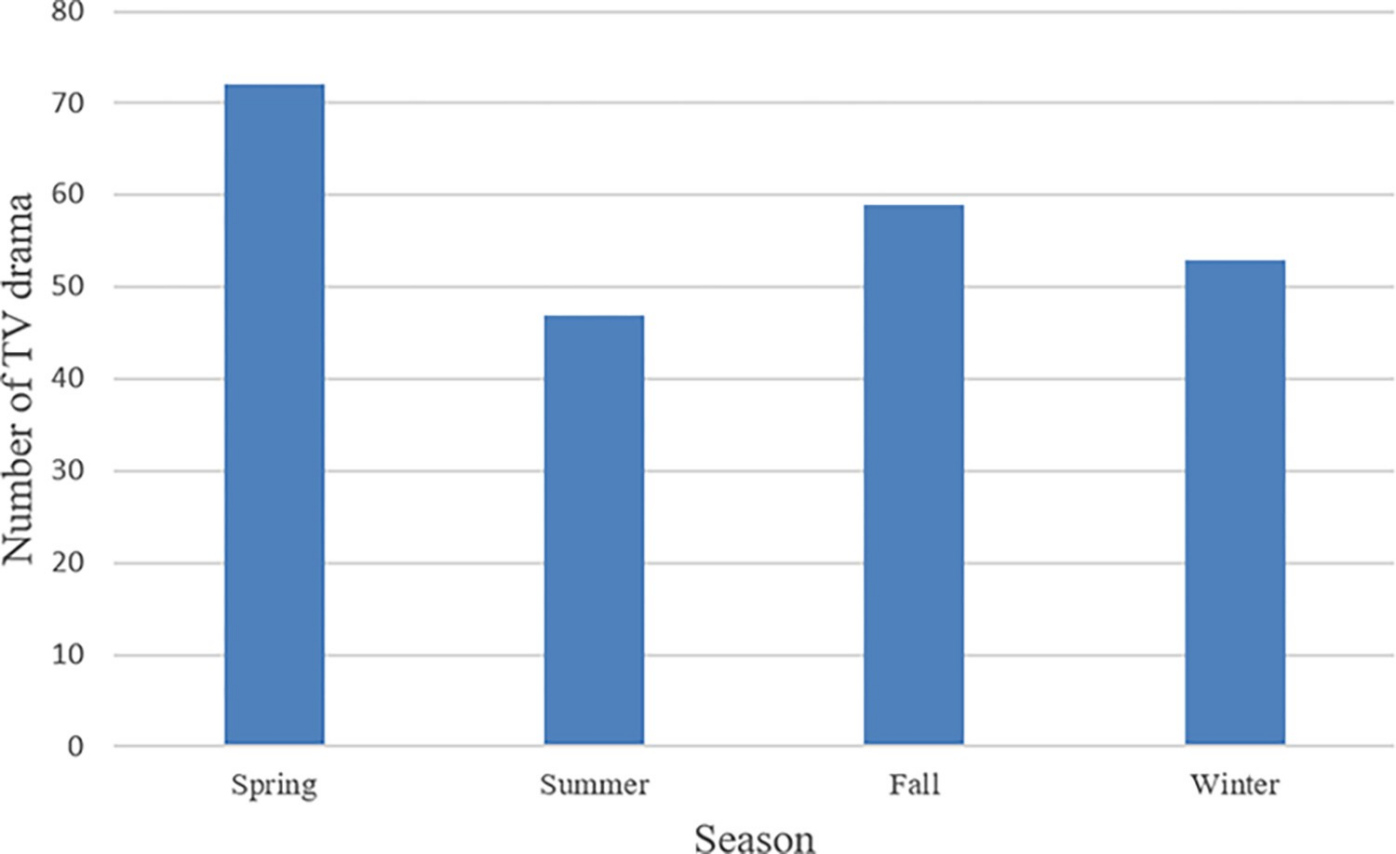
The number of highly rated dramas by season.

After clustering, the threshold used to determine drama ratings as high or low was 12.66%. Fig 6 shows the number of highly rated TV dramas between 2003 and 2020 according to the broadcast year. The year 2005 has the most highly rated TV dramas, with 27. Since 2015, the number has not exceeded 10. Fig 7 shows the histogram of highly rated groups according to the broadcast season. The highest and lowest numbers appear in spring and summer, respectively.

**Fig 8:**
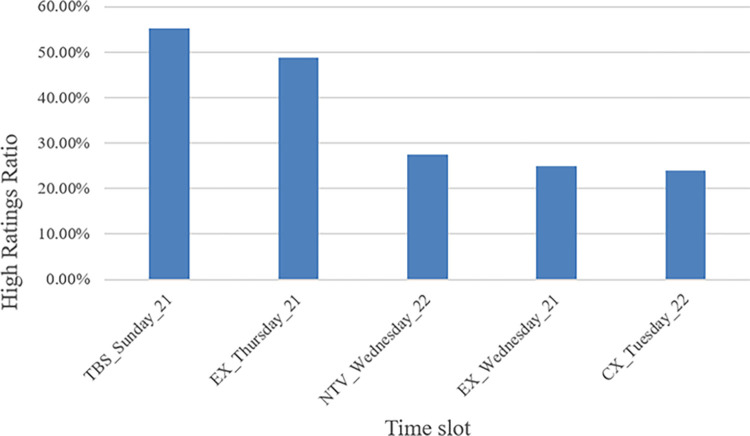
Highly rated scheduling slots.

**Fig 9:**
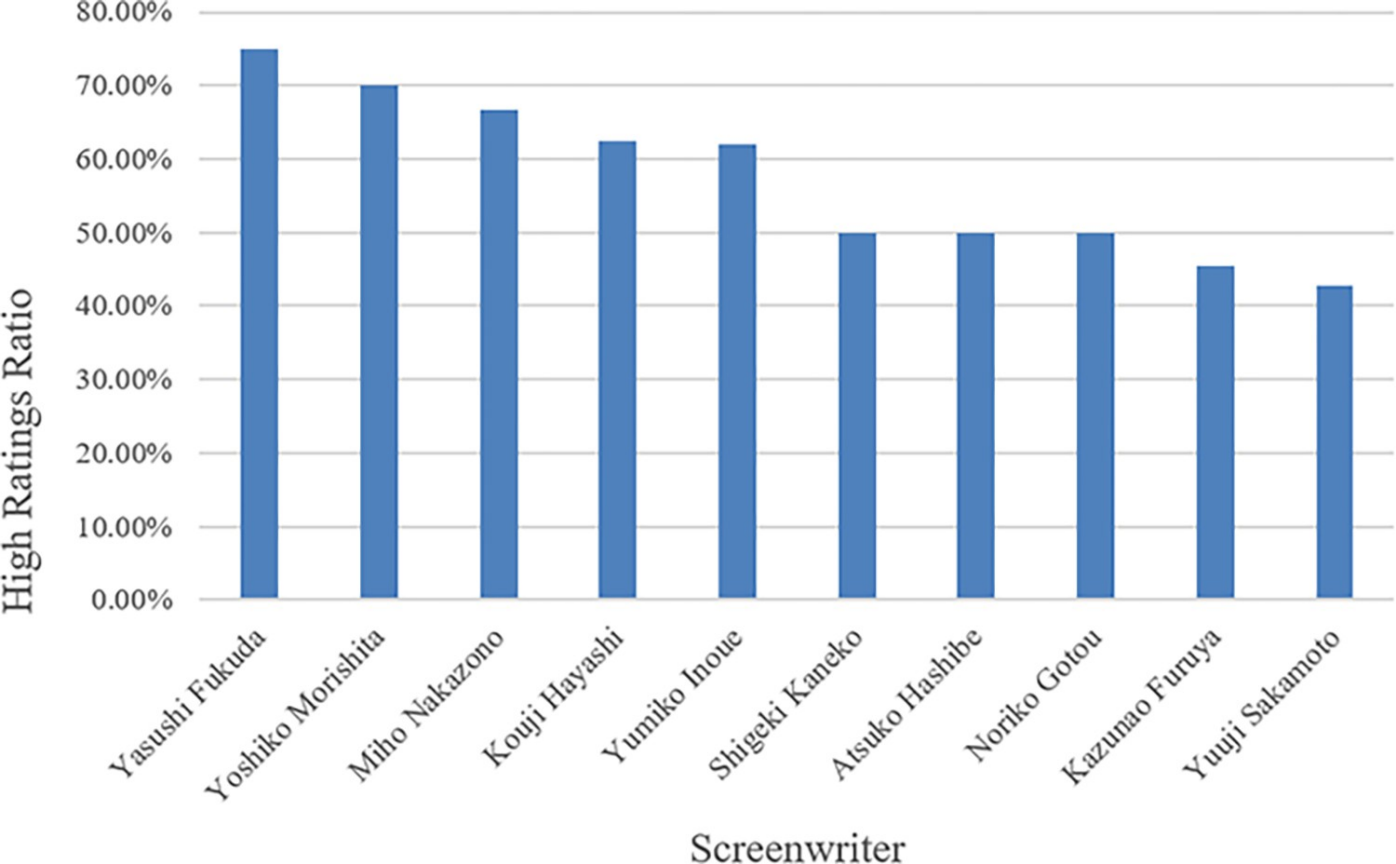
Highly rated screenwriters.

Fig 8 shows the top five scheduled slots with a high ratio of highly rated dramas, including TBS TV Sunday 9 pm, Asahi TV Thursday 9 pm, Nippon TV Wednesday 10 pm, Asahi TV Wednesday 9 pm, and Fuji TV Tuesday 10 pm. Fig 9 shows the top 10 screenwriters with more than 10 works in the dramas that have the highest percentage of episodes in highly rated groups. Yasushi Fukuda has the most highly rated episodes at 75.00%.

**Fig 10:**
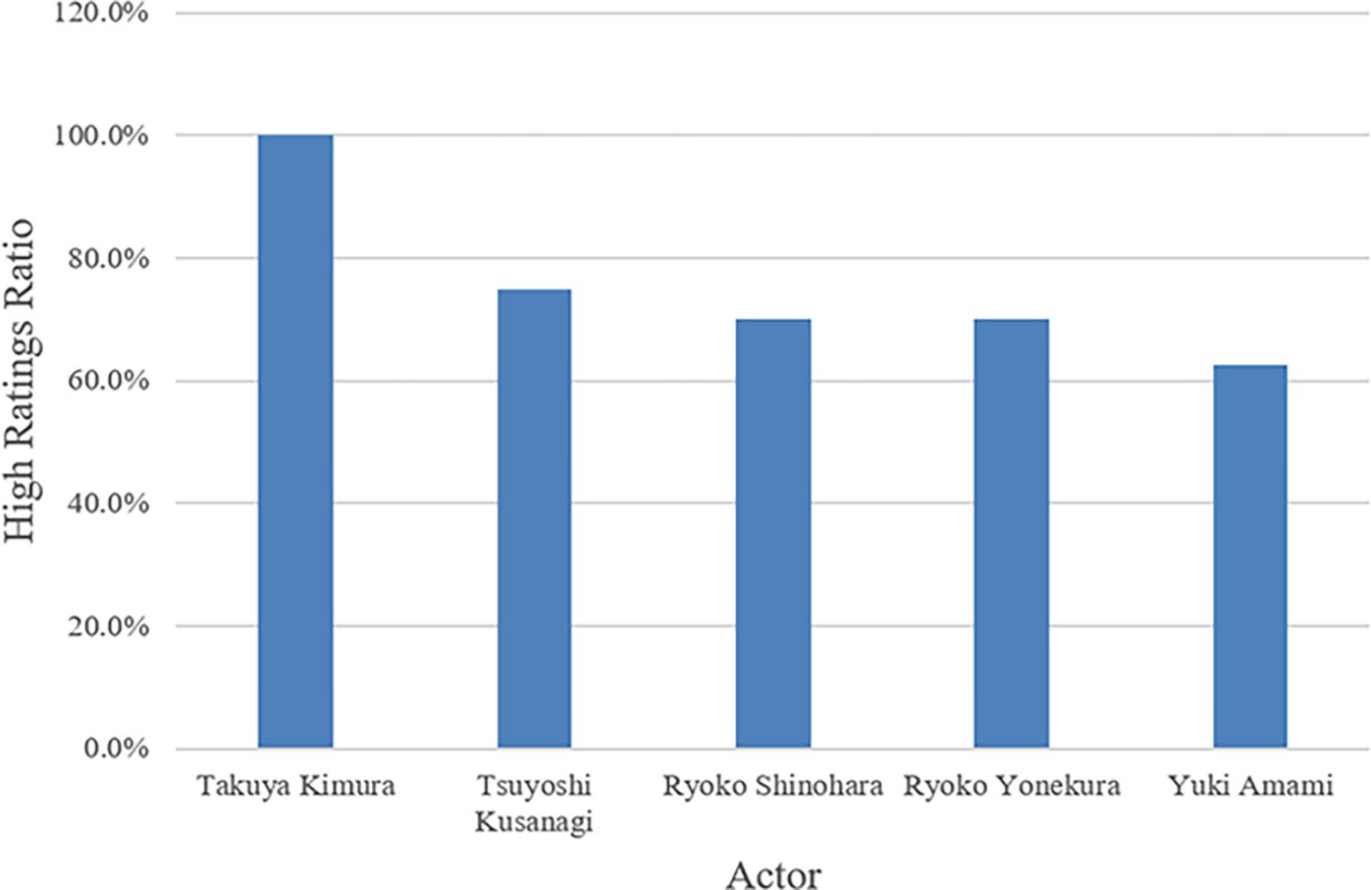
Highly rated leading actors.

Fig 10 shows the top five leading actors ranked by the percentage of highly rated dramas which they acted in. The minimum criterion was set to more than five dramas in the dataset for each actor. Takuya Kimura has a 100.00% ratio in highly rated dramas.

The top five genres with a high percentage of highly rated dramas are medical, history, action, sports and emotion. Medical drama has the highest percentage at 50.00%. Among highly rated dramas, 129 are original works and 102 are adaptations. Only 18.00% of sequels are in the highly rated group.

### Numerical prediction model

The proposed drama metadata were used as features in the rating prediction. In the experiment, the computational environment including Intel^®^ Core^™^ i9-9980HK CPU @ 2.40 GHz, 32 GB main memory, and an NVIDIA GeForce RTX 2080 with 8 GB of GPU memory. Python 3.9.13 with sklearn = 1.0.2 and MATLAB 2021b (MathWorks, Natick, MA, USA) were the software environment. During the training, tenfold cross-validation was used to provide generalizability. The whole dataset was randomly divided into 10 subsets of approximately the same size. One subset was picked to validate the model after it was trained on the remaining nine subsets. After 10 iterations of training and validation, the final result is obtained by averaging. Table 2 shows that a single feature can achieve predictive accuracies between 68.9% and 72.5% in the four classifiers. Only using more features can obtain better results. Based on RF, combining the original 10 features produced a ratings prediction accuracy of 74.90%. Using a scheduled slot instead of a TV station, day of week, and broadcast time in the combination, the accuracy can be improved to 75.80%.

**Table 2:** Performance comparisons of different features and classifiers.

Classifier	NB [26]	ANN [27]	SVM [28]	RF [29]
Year	71.10%	72.50%	71.10%	72.50%
Season	71.10%	71.10%	71.10%	71.10%
TV station	70.00%	71.10%	71.10%	71.10%
Day of week	71.10%	71.10%	71.10%	71.10%
Broadcast time	71.10%	71.10%	71.10%	71.10%
Genre	71.10%	68.90%	70.10%	69.80%
Screenwriter	71.50%	71.10%	71.10%	69.50%
Original work	71.10%	71.10%	71.10%	71.10%
Sequel	71.10%	71.10%	71.10%	71.10%
Cast	71.10%	71.10%	71.10%	71.10%
Scheduled slot (TV station & day of week & broadcast time)	71.10%	70.80%	70.50%	71.20%
Original 10 features	71.20%	71.10%	71.10%	74.90%
Scheduled slot +7 features	70.80%	71.10%	71.10%	75.80%

Azure Face was used in extracting facial features in drama posters. To have a better image resolution and interpretation, poster images were enlarged 3 times in equal proportions. Using the extracted facial features alone obtained 61.30% to 71.10% predictive accuracy. Nevertheless, combining facial features with scheduled slots and the other seven features resulted in an accuracy of 77.01%, as shown in Table 3.

**Table 3:** Comparison of the accuracy of using facial features in different classifiers.

Classifier	NB [26]	ANN [27]	SVM [28]	RF [29]
Facial feature	70.20%	61.30%	71.10%	68.00%
Face+scheduled slot+7 features	71.40%	71.10%	71.10%	77.10%

Using CNNs in model training and validation with datasets split by 80.00% and 20.00%, respectively, the accuracies of AlexNet [30], Inception V3 [31], ResNet 152 [32], and DenseNet 201 [33] were 61.01%, 71.07%, 71.07%, and 71.70%.

## Discussion

Numerical drama metadata and poster features were quantified and combined in the experiment to predict ratings. Each single feature had an accuracy of approximately 70.00% using the RF classifier. The best accuracy of 77.10% was achieved by combining all the features together in the RF classifier. Each feature was helpful for estimating the ratings. However, only combining them together can generate the best predictive performance. The scheduled slot as an integrated feature was transformed according to known viewer habits. The result agrees with a previous study [14] that Japanese audiences may be accustomed to watching TV dramas in a fixed scheduled slot. Broadcast in highly rated scheduled slots, these dramas are expected to have stable ratings. Advertisers investing in such scheduled slots may reach more audiences and obtain more revenue. A similar feature is the year of release. The 18-year time span of the collection period is large. It reveals that the number of TV dramas with high ratings declined from 2011 to 2019, and the ratings of TV dramas showed a decreasing trend. From this perspective, we can hardly expect higher ratings in the future production of TV dramas. A larger time slot feature is the season. Fewer highly rated TV dramas occurred in summer than in the other three seasons, which can be attributed to some possibilities. Summer is the season when many large sports events are held and TV dramas are often stopped or delayed due to the broadcast of sporting events, which affects the viewing habits for TV dramas. In addition, most Japanese people travel during summer vacation and do not watch TV at home.

The leading actors also contribute to the ratings. Takuya Kimura, Tsuyoshi Kusanagi, Ryoko Shinohara, Ryoko Yonekura and Yuki Amami are the star actors with high appeal. Having these actors as leads may increase the ratings of TV dramas. The situation is similar with screenwriters who create highly rated episodes. Medical dramas are the most likely to achieve high ratings among all genres. A study stated that being sick or attending a medical appointment is an experience that most people have personally experienced and is more likely to resonate than other subjects. The highly professional nature of medical practices and the surgical procedures performed by medical personnel are unfamiliar to most people, and this relationship with people’s lives is one of the attractions of watching medical dramas. In addition, another major reason is the aging of TV viewerships. Older audiences are more concerned about their future health than youth and romance dramas; thus, medical dramas often have high ratings.

In terms of predicting ratings using CNNs on posters, using pretraining on ImageNet and DenseNet201 obtained an accuracy of 71.70% from 800 posters. Compared with the literature reported by Zhou [17], the pretrained model that was also based on ImageNet used 3807 movie posters on IMDb to classify the box office returns into 6 levels and obtained an accuracy of 63.15%. The methodologies are similar and the results are also similar. That is, it is still challenging to obtain a higher performance, but posters still have a meaningful effect on increasing accuracy in the prediction.

## Conclusion

This study used drama metadata and posters to realize that drama, through synchronization with social phenomena, allows the audience to resonate with the characters. The rating prediction can be helpful for understanding viewing indicators, which are the standard of advertising revenue and subsequent economic efficiency in the surrounding areas. Using numerical features including broadcast year, broadcast season, TV stations, day of the week, broadcast hour, genre, screenwriters, status as original work or sequel, cast and facial features from 800 Japanese TV dramas, RF reached an accuracy of 77.10%, revealing the usefulness of facial information. Drama posters can also provide some clues and result in 71.70% accuracy by DenseNet201. The statistical analysis also shows the factors that affect ratings and the cultural connotations behind these factors. In the future, the content of dramas, including voices, frames, and subtitles, may be quantified to present more details about the stories themselves. Additionally, budget may be related to the sophistication and plot arrangement of TV dramas. These features would be helpful in rating prediction and thus bring more possibilities to planning a high-performing drama.

## Supporting information

S1 Data (RAR)
